# The endogenous cardiotonic steroid Marinobufagenin and decline in estimated glomerular filtration rate at follow-up in patients with arterial hypertension

**DOI:** 10.1371/journal.pone.0212973

**Published:** 2019-02-28

**Authors:** Martin H. Keppel, Grzegorz Piecha, Winfried März, Janne Cadamuro, Simon Auer, Thomas K. Felder, Cornelia Mrazek, Hannes Oberkofler, Christian Trummer, Martin R. Grübler, Verena Schwetz, Nicolas Verheyen, Marlene Pandis, Valentin Borzan, Elisabeth Haschke-Becher, Andreas Tomaschitz, Stefan Pilz

**Affiliations:** 1 University Institute for Medical and Chemical Laboratory Diagnostics, Paracelsus Medical University, Salzburg, Austria; 2 Department of Nephrology, Transplantation and Internal Medicine, Medical University of Silesia, Katowice, Poland; 3 Synlab Academy, Mannheim, Germany; 4 Medical Clinic V (Nephrology, Hypertensiology, Rheumatology, Endocrinology, Diabetology), Medical Faculty Mannheim, University of Heidelberg, Heidelberg, Germany; 5 Clinical Institute of Medical and Chemical Laboratory Diagnostics Medical, University of Graz, Graz, Austria; 6 Department of Endocrinology and Diabetology, Medical University of Graz, Graz, Austria; 7 Department of Cardiology, Swiss Cardiovascular Center Bern, Bern University Hospital, University of Bern, Bern, Switzerland; 8 Department of Cardiology, Medical University of Graz, Graz, Austria; 9 Bad Gleichenberg Clinic, Bad Gleichenberg, Austria; International University of Health and Welfare, School of Medicine, JAPAN

## Abstract

**Background:**

Marinobufagenin (MBG) is an endogenous cardiotonic steroid (CTS) that inhibits the Na+/K+-ATPase. Human MBG is significantly increased in end-stage renal disease and immunization against MBG attenuates cardiovascular fibrosis in a rat model of uremic cardiomyopathy. Mineralocorticoid antagonists (MRA) block MBG binding sites and decrease proteinuria in chronic kidney disease (CKD) patients. We therefore aimed to investigate the association of MBG and albuminuria, as a marker of renal damage, as well as MBG and decline of glomerular filtration rate (GFR).

**Methods:**

The Graz endocrine causes of hypertension (GECOH) study is a single center study of adults routinely referred for screening of endocrine hypertension. Plasma MBG was measured by an enzyme-linked immunoassay, and in a post-hoc analysis, follow-up creatinine levels were obtained. Patients with proteinuria >3.5g/day at baseline were excluded from further evaluation.

**Results:**

We measured MBG concentrations in 40 hypertensive subjects and excluded one patient due to pre-existing proteinuria. Plasma MBG was significantly correlated with albuminuria (Spearman *ρ* = .357; p = .028) and proteinuria (*ρ* = .336; p = .039). In linear regression analysis, the association remained significant after adjustment for age, sex, and BMI (β = .306; p = .036), and for mean systolic blood pressure (β = .352; p = .034). In follow-up analyses (N = 30), MBG was significantly associated with decline in GFR after adjustment for time-to-follow-up (β = -.374; p = .042).

**Conclusion:**

The findings suggest that MBG plasma concentrations were associated with albuminuria as well as decline in kidney function. Whether MBG predicts hard renal endpoints warrants further investigations.

## Introduction

Chronic kidney disease (CKD) is one of the most burdensome and frequent medical conditions. In general populations, CKD prevalence of all five KDIGO stages is 13.4%, and of KDIGO stages three to five is 10.6%.[1] CKD is regarded as an accelerator of cardiovascular (CV) risk and an inverse relationship between CV risk and glomerular filtration rate (GFR) exists.[1] KDIGO guidelines emphasize risk stratification according to grades of albuminuria to minimize false identification of CKD.[2] However, although albuminuria may be a valid screening tool for renal impairment and serve as a prognostic factor for CV risk [3], its prognostic value for further GFR decline is still a matter of discussion.[4]

Mineralocorticoid receptor antagonist (MRA) therapy has been suggested to mitigate renal fibrosis.[5,6] MRA decreased proteinuria in CKD subjects by up to 23% to 61%[7,8] and lowered biomarkers associated with CKD progression in rats, e.g. tissue expression of Type I and III collagen [9]. MRA therapy may delay CKD progression over the long term [10], but studies concerning MRA therapy and improvement of hard kidney endpoints are pending so far.[11]

Marinobufagenin (MBG) is an endogenous cardiotonic steroid (CTS), all of which are inhibitors of the sodium-potassium adenosine triphosphatase (Na+/K+-ATPase), also called digitalis-like factors. By chemical structure, MBG belongs to bufadienolides.[12] First described in toads, MBG can be found in high concentrations in the skin of amphibians, where it is hypothesized to be integral to water and electrolyte homeostasis. Amphibian MBG concentrations respond appropriately to changes in environmental salinity whereas in humans, increased plasma concentrations of bufadienolides are associated with excessive salt and fluid accumulation.[13] MBG plasma concentrations are increased by sodium loading and in turn increase natriuresis by a pressure induced mechanism via vasoconstriction and by direct effects on the renal tubule. In line with this notion, elevated concentrations of MBG were reported for a variety of clinical conditions associated with body fluid volume expansion, such as congestive heart failure (CHF), end-stage renal disease (ESRD), hypertension (HTN), renal ischemia, and preeclampsia.[14] We showed that plasma MBG concentrations were higher in patients with primary aldosteronism compared to essential hypertension.[15] Abnormalities in renal sodium handling have been proposed as a major cause of arterial hypertension and cardiovascular remodeling. In rats, MBG infusion for four weeks significantly increased plasma aldosterone concentrations and systolic blood pressure. Infusion of MBG in rats also caused renal fibrosis, and passive immunization against MBG attenuated renal fibrosis and improved renal function.[14,16] MRA therapy was shown to occupy CTS binding sites preventing pro-fibrotic MBG effects.[17]

The circulating concentrations of MBG are significantly increased in virtually all patients undergoing dialysis for ESRD.[18–21] Higher MBG immunoreactivity has been associated with worse all-cause mortality in hemodialyzed patients.[22] Endogenous CTS served as biomarkers for acute kidney injury during elective cardiac surgery.[23] Furthermore, MBG may be responsible for many of the clinical features of experimental uremic cardiomyopathy, suggesting that MBG may be at least a potential marker of renal impairment and of progression of chronic kidney disease (CKD).[17]

We therefore evaluated the relation of plasma MBG and albuminuria, as a clinical marker of kidney damage, in patients with arterial hypertension in a post-hoc analysis of the Graz endocrine causes of hypertension (GECOH) study. In addition, we assessed the association of plasma MBG concentrations and decline of estimated GFR at follow-up.

## Materials and methods

### Study design and ethics approval

The Graz endocrine causes of hypertension (GECOH) study is a single center study at a tertiary care center that comprises adult patients (aged ≥18 years), who have routinely been referred to the outpatient clinic for screening for endocrine hypertension at the Division of Endocrinology and Diabetology, Medical University of Graz, Austria. The study protocol has previously been published elsewhere. [24] The primary aim of this study was the diagnostic accuracy of the aldosterone to renin ratio for the diagnosis of primary aldosteronism. In brief, main inclusion criterion was diagnosis of arterial hypertension and the main exclusion criterion was an ongoing MRA therapy (i.e. spironolactone, canrenoate, eplerenone) up to four weeks before study entry. The GECOH study was approved by the Ethics Committee at the Medical University of Graz (EK number 20–109 ex 08–09) and all participants gave written informed consent to follow-up analyses. All analyses are adhering to the recommendations of the Declaration of Helsinki.

### Measurements of clinical variables

In a first baseline visit, arterial blood pressure (BP) was measured according to the method of Korotkoff after five minutes at rest and a sphygmomanometer with an appropriate cuff. Systolic and diastolic BP values were measured on both arms and the mean values were recorded. Height and weight were determined wearing light clothes and no shoes. Detailed methods about diagnostic criteria of primary aldosteronism (PA) have been described previously.[25]

### Measurements of biochemical variables

At a first baseline visit, venous blood sampling was performed according to current guidelines.[26] Blood was drawn in the morning (8:00–11:00 A.M.) after the patients had been seated for ten minutes, and midstream spot urine samples were collected. Laboratory and clinical assessments were performed after at least twelve hours of fasting. Patients were on an unrestricted Western diet and were advised to avoid both smoking and any medication intake in the morning before blood sampling.

After a maximum of five-year storage at -80°C in aliquots of 1 mL without additional freeze-thaw cycle, plasma MBG was measured at the Department of Nephrology, Transplantation and Internal Medicine, Medical University of Silesia, Katowice, Poland, following extraction with C-18 columns (Waters Inc. Cambridge, MA, USA).[27] Detailed methodology of MBG measurement have been described elsewhere.[22] In short, plasma MBG was determined by an enzyme-linked immunoassay (ELISA). Respective plates were coated with MBG-bovine serum albumin conjugate at a dose of 5 ng/well. Anti-MBG mouse monoclonal antibody (4G4, titer 1:1000) was used (100 μL/well) followed by biotin-labeled anti-mouse secondary antibody (Abcam, Cambridge, UK) and streptavidin-alkaline phosphatase conjugate (Perkin Elmer, Waltham, MA, USA). FirePhos (KPL Inc., Gaithersburg, MD, USA) substrate was used and the absorbance read at 480 nm wavelength.[28] Intra- and inter-assay coefficient of variation (CV) were 6.5–8.6 and 8.3–13.6%, respectively.[22] Parameters of hematology, clinical chemistry and urinalysis were determined the same day of collection by routine laboratory procedures at the Medical University of Graz, Austria. In a second study baseline visit, blood gas analytes were obtained and 24-hour urine samples collected.

Plasma creatinine was measured at the Clinical Institute of Medical and Chemical Laboratory Diagnostics Medical, University of Graz, Graz, Austria, using the Jaffe method, with a calibration traceable to an isotope dilution mass spectrometry (IDMS) reference measurement procedure (Cobas Creatinine Jaffé Gen.2 compensated, Roche Diagnostics, Mannheim, Germany).

### Follow-up variables and retrospective data interpretation

In January 2018, we retrospectively obtained available plasma creatinine concentrations of participants from the local hospital information system, where only the single most recent follow-up value was used for the present analysis. Estimated glomerular filtration rate (GFR, ml/min/1.73m^2^) at baseline and follow-up was determined using the CKD-EPI (Chronic Kidney Disease Epidemiology Collaboration) formula.[29]

For all present analyses, we included patients with baseline plasma MBG concentrations available. Patients with proteinuria >3.5g/day at baseline as a sign of existing or diagnosed progressed kidney damage (i.e. diabetic nephropathy, renal artery stenosis, vasculitis or glomerulonephritis) were excluded from further analyses.

### Statistical analyses

Depending on their distribution, continuous data are either presented as means ± SD (normally distributed variables) or as medians with interquartile ranges (skewed variables). Categorical variables are presented as percentages. To test for normality we used Shapiro-Wilk tests. Group comparisons were performed by analysis of variance (ANOVA), Kruskal-Wallis test by ranks or chi-squared test for linear trends, where appropriate. Pearson’s *r* coefficients were computed to assess the association between parametric variables, Spearman’s *ρ* coefficients for non-parametric variables. Linear regression analyses were further used to account for the influence of potential confounding variables. To lower risk of over-fitting, in cox regression analyses, MBG concentrations were allocated into three equally sized groups and the hazard of grouped plasma MBG concentrations on falling into a lower CKD class according to KDIGO guidelines [2] at follow-up was estimated. The 95% confidence intervals were estimated by profile likelihood with regard to the small sample size. To estimate the average GFR decline per year per 0.1 nmol/L increase in plasma MBG, we further obtained estimates of linear regression analysis, after additionally excluding patients with follow-up time <1 year, as short-term fluctuations in creatinine would have an outweigh impact on the association. Data were analyzed using SAS Studio University Edition version 2.5 (SAS Institute Inc., 100 SAS Campus Drive Cary, NC, U.S.A). A p-value ≤ 0.05 was considered statistically significant.

## Results

Between February 2009 and December 2012, 233 white study participants were enrolled at the outpatient clinic of the Division of Endocrinology and Diabetology, Medical University of Graz, Austria. Plasma marinobufagenin (MBG) concentrations were available in 40 patients, of which 11 patients were diagnosed with primary aldosteronism (PA) and 29 patients with essential hypertension (after exclusion of PA). One patient was excluded from the present analysis due to pre-existing diabetic nephropathy and excessive proteinuria >3.5g/day at baseline.

Overall and disease-specific baseline characteristics of remaining study participants (N = 39) are shown in Table 1. After classification into three MBG groups, we noticed a non-significant trend of increased albuminuria and proteinuria with increasing groups of plasma MBG concentrations (Table 2). Plasma MBG concentrations were not significantly different between men (median 0.43; interquartile range [IQR] 0.28–0.75) and women (median 0.67; IQR 0.33–0.74; two-sided difference p = .410).

**Table 1:** Summary characteristics of participants.

	Overall	Essential hypertension	PA	p
**Age (yrs)**	51.8 +/- 13.0	52.6 +/- 14.0	49.7 +/- 10.6	.528
**Sex (% female)**	56%	54%	64%	.725
**BMI (kg/m**^**2**^**)**	29.5 +/- 5.4	29.4 +/- 4.7	29.8 +/- 7.5	.836
**GFR (ml/min/1,73m**^**2**^**)**	80.6 +/- 15.3	78.9 +/- 15.0	85.5 +/- 15.8	.205
**MBG (nmol/L)**	0.53 +/- 0.25	0.46 +/- 0.24	0.74 +/- 0.16	.006 *
**Systolic RR (mmHg)**	163 +/- 24	157 +/- 22	178 +/- 22	.014 *
**Urinary albumin (mg/L)**	17.5 (0–37.2)	11.0 (0–29.0)	20.0 (13.0–50.0)	.180
**Urinary protein (mg/L)**	95.5 (60.5–138.8)	90.0 (58.0–126.0)	125.0 (82.0–141.0)	.139

Overall and disease-specific summary characteristics of participants (N = 39). We included 11 patients with primary aldosteronism (PA) and 28 essential hypertensive controls. Yrs = years; BMI = Body mass index; GFR = estimated glomerular filtration rate (CKD-EPI); MBG = plasma marinobufagenin; RR = blood pressure;

* = significant.

**Table 2:** Characteristics of study participants according to MBG groups.

	N	Age (yrs)	Sex (% female)	BMI (kg/m^2^)	GFR (ml/min/1, 73m^2^)	Systolic RR (mmHg)	Spot urine albumin (mg/L)	Spot urine protein (mg/L)	Spot urine sodium (mmol/L)	Spot urine sodium-creatinine ratio	24 hour sodium excretion (mmol/24h)
**MBG** **<= .33 nmol/L**	13	49.9 +/- 17.1	69%	30.8 +/- 5.5	84.7 +/- 16.5	160 +/- 29	12.2 +/- 13.7	78.5 +/- 33.4	129 (100–171)	0.94 (0.82–1.52)	142 (129–289)
**MBG** **.34–.68 nmol/L**	13	53 +/- 10.3	42%	28.9 +/- 5.2	76.0 +/- 15.2	161 +/- 19	43.9 +/- 66.8	136 +/- 122.1	83 (75–129)	0.97 (0.36–1.33)	144 (108–233)
**MBG** **.69 + nmol/L**	13	52.4 +/- 13.0	54%	28.5 +/- 5.7	80.8 +/-14.0	168 +/- 23	45.5 +/- 53.6	151.5 +/- 106.5	75 (51–123)	0.60 (0.36–1.04)	118 (93–174)
**p**		.817		.535	.255	.661	.173	.108	.044*	.460	.569

Data are presented as means +/- standard deviation or medians with interquartile ranges in brackets. yrs = years; BMI = Body mass index; GFR = estimated glomerular filtration rate (GFR-EPI); MBG = plasma marinobufagenin; RR = blood pressure;

* = significant.

Plasma MBG concentrations were significantly correlated with albuminuria (Spearman *ρ* = .357; p = .028) and proteinuria (*ρ* = .336; p = .039), but not with GFR at baseline, or systolic BP at baseline. In linear regression analysis, the association remained significant after adjustment for possible confounders, including age, sex, and BMI (β = .306; p = .036), and for mean systolic blood pressure (β = .352; p = .034). However, after adjustment with GFR at baseline, significance was lost (β = .281; p = .074).

In a sensitivity analysis, the correlation between plasma MBG concentrations and albuminuria at baseline was still evident in patients with essential hypertension (*ρ* = .428; p = .026), but not in patients with PA (*ρ* = -.166; p = .627).

Of 39 patients at baseline, follow-up plasma creatinine concentrations were available in 30 patients. Characteristics of study participants at follow-up are shown in Table 3, median follow-up time was 4.25 years (IQR 2.4–7.3 years).

**Table 3:** Characteristics of study participants according to MBG groups at follow-up.

	N	Sex (% female)	Time-to-follow-up (yrs)	GFR (ml/min/1,73m^2^) *	GFR difference (ml/min/1,73m^2^)	PA (% prevalence)
**MBG <= .33 nmol/L**	6	100%	6.7 (2.0–7.1)	109.0 +/- 11.5	- 1.3 +/- 10.5	17%
**MBG .34–.68 nmol/L**	12	42%	3.1 (2.3–6.7)	72.6 +/- 23.5	- 0.7 +/- 9.7	25%
**MBG .69 + nmol/L**	12	50%	4.3 (2.8–6.8)	70.0 +/-30.8	- 10.8 +/- 11.8	58%

yrs = years; GFR = estimated glomerular filtration rate (CKD-EPI); PA = Primary aldosteronism; MBG = plasma marinobufagenin;

* = significant.

**Fig 1:**
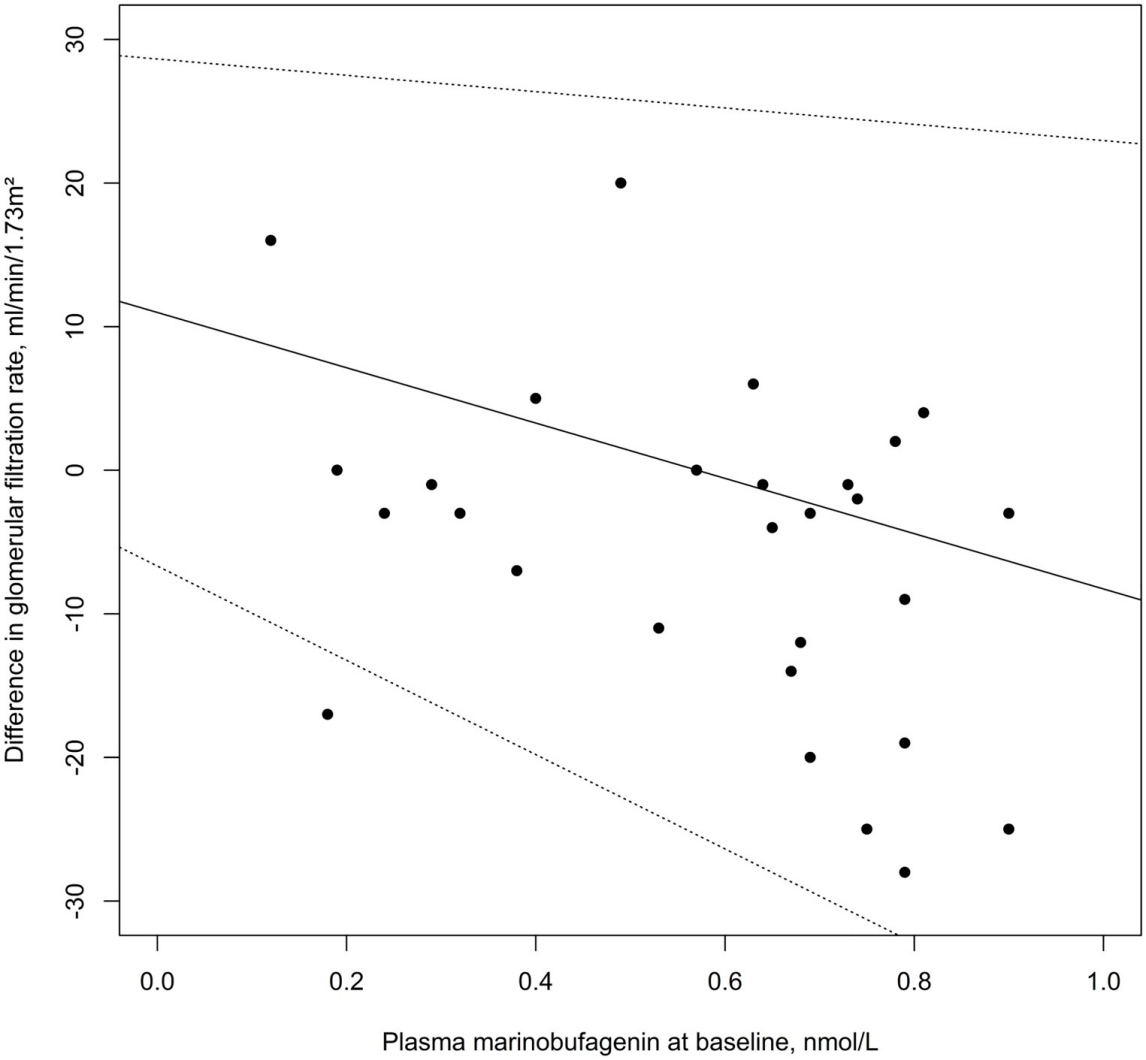
Marinobufagenin concentrations and decline in estimated glomerular filtration rate. Scatterplot of plasma marinobufagenin (MBG) concentrations at baseline visit (in nmol/L) and difference in estimated glomerular filtration rate (GFR, in ml/min/1.73m^2^) at follow-up: Regression line (solid line) with 95% confidence interval (dotted lines) after adjustment for time-to-follow-up.

In follow-up analyses, the correlation of MBG and decline in GFR showed borderline non-significance (*ρ* = -.361; p = .052). In linear regression analysis, the association turned significant after adjustment for time-to-follow-up (β = -.374; p = .042; Fig 1), for age and sex (β = -.430; p = .042), for baseline variables hematocrit (β = -.420; p = .039), plasma uric acid (β = -.471; p = .015), plasma phosphorus (β = -.391; p = .049), spot urine sodium (β = -.503; p = .017), and spot urine sodium-to-creatinine ratio (β = -.407; p = .047). Although not significant, the association of MBG and decline in GFR still showed a trend for significance after adjustment for baseline variables mean plasma calcium (β = -.375; p = .053), systolic BP (β = -.361; p = .056) and albuminuria (β = -.372; p = .063), but not for present diabetes mellitus type I or II at baseline (N = 6; β = -.339; p = .074), history of smoking at baseline (β = -.319; p = .107), and fasting glucose at baseline (β = -.301; p = .106), LDL at baseline (β = -.363; p = .081), bicarbonate (β = -.165; p = .442), and 24-hour sodium excretion (β = -.375; p = .077). The adjustment for diagnosis of primary aldosteronism had no impact magnitude of basic correlation coefficients, but association remained non-significant (β = -.316; p = .113). Adjustment for different antihypertensive drug categories also turned the association of MBG and decline in GFR to insignificance mostly: Angiotensin-converting enzyme inhibitors (ACEI; N = 14; β = -.354; p = .064), angiotensin II receptor blockers (ARB; N = 7; β = -.365; p = .065), beta-blocker (BB; N = 25; β = -.404; p = .033), Calcium-channel blockers (CCB; N = 17; β = -.372; p = .050), diuretics (N = 15; β = -.362; p = .056), and mineralocorticoid-receptor antagonists (MRA; N = 10; β = -.241; p = .613).

In sensitivity analyses, significance of the correlation of plasma MBG and decline in GFR was lost for both patients with essential hypertension (β = -.334; p = .162) and PA (β = -.174; p = .608). No association was found for albuminuria at baseline and decline in GFR (*ρ* = -.124; p = .520). MBG correlated with spot urine sodium (*ρ* = -.335; p = .040), but not with spot urine sodium-to-creatinine ratio (*ρ* = -.154; p = .376), nor 24-hour sodium excretion (*ρ* = -.036; p = .851).

In cox regression, the hazard ratio of increasing plasma MBG concentrations for the risk of falling into a lower CKD class at follow-up according to KDIGO guidelines was 3.6 (95% confidence interval [CI] 1.2–21.1; p = .063). Eventually, an increase in 0.1 nmol/L plasma MBG at baseline was associated with a decrease of 2.75 ml/min/1.73m^2^ GFR (95% CI -2.83 –-2.66; p = .024) per year during follow-up.

## Discussion

In this study, we demonstrated a significant association between plasma marinobufagenin (MBG) concentrations and albuminuria, a marker of renal damage. In addition, we could demonstrate that elevated plasma MBG concentrations correlate with a decline in estimated glomerular filtration rate (GFR) at follow-up. The results indicate that, for hypertensive patients, MBG could serve as a potential marker for kidney impairment at follow-up. In subgroup analyses, the association was evident in patients with essential hypertension, although we showed that MBG concentrations are higher in PA patients than in patients with essential hypertension.[15] The selection of potential confounding variables was based on known risk factors for CKD progression.[30] However, the small sample size did not allow a fully adjusted regression model without inflating of coefficients and limited further investigation of potential confounding variables.

MBG concentrations are elevated in patients with end-stage renal disease (ESRD) and MBG was suggested to mediate cardiovascular remodeling in uremic cardiomyopathy. In rats with experimental renal disease, MBG was associated with renal fibrosis and passive immunization against MBG attenuated progression of fibrosis as well as improved renal function.[14] To our knowledge, the present study is the first one that shows that in humans elevated plasma MBG concentrations are associated with subsequent decline in GFR, irrespective of pre-existing CKD. Our data therefore support the notion that elevated MBG concentrations may already precede renal impairment rather than being a mere consequence of it.

MBG usually binds to Na+/K+-ATPase and dissociates very slowly, so that the cell induces endocytosis of the blocked Na+/K+-ATPase. The Na+/K+-ATPase resides ubiquitously at the plasmamembrane barrier of various cell types including human cardiomyocytes, vascular smooth muscle cells, cells of the adrenal gland and epithelial cells of the proximal tubule. Besides the basic function of Na+/K+-ATPase to maintain a gradient of sodium and potassium concentrations across the plasmamembrane barrier, Na+/K+-ATPases take part in signaling pathways of various cell lines.[31] The Na+/K+-ATPase then acts as co-factors of several signaling proteins, like the Epidermal Growth Factor Receptor (EGFR) in human muscle cells and modulates channel voltage sensitivity in neurons.[32] Through the Na+/K+-ATPase, MBG transforms the protein levels and nuclear localization of the transcription factor Snail in the tubular epithelia cells, promoting cardiovascular and renal fibrosis.[31] During a sodium-rich diet, MBG induces endocytosis of the proximal tubular Na+/K+-ATPase and reduces renal sodium reabsorption, increasing sodium excretion. Subsequently the overall number of available Na+/K+-ATPase decreases, which in turn may constrain the renal capacity to adequately respond to further fluctuations in sodium and water intake. A depletion of Na+/K+-ATPase may then promote fluid retention and volume expansion.[31] MBG was shown to be involved in the pathogenesis of vascular fibrosis and such remodeling was reduced by immunoneutralization of MBG.[33] MBG-induced vascular fibrosis is a likely target for mineralocorticoid receptor antagonist (MRA) therapy as MRA desensitize Na/K-ATPase to pro-fibrotic effects of MBG and restore Na/K-ATPase activity in the presence of MBG.[16,17]

MRA therapy was shown to delay CKD progression over the long term.[10] Still, MRA are underused in clinical practice. One reason for the underuse is an unfavorable set of side effects that comes in the form of increased risk for hyperkalemia, particularly when GFR is low, as well as an antiandrogenic profile. Other reasons may concern the conflicting results of meta-analyses, including inconsistent renal outcomes [34,35] and indicating that not every CKD patient may benefit from MRA therapy.[36] However, many of the studies included covered only a short treatment time of several weeks and may not be able to detect long-term effects of MRA therapy. Study quality varied across studies, allocation concealment and blinding were often deficient, and a minor quantity of studies performed intention-to-treat analysis.[11] Finally, as CKD may be a complex set of different pathologies, Na+/K+-ATPase disturbances might not be involved in the pathogenesis of every sub-entity of CKD. MRA therapy then might not be effective for the whole group of CKD patients.

MBG may not only mediate renal fibrosis but may help to detect individuals at risk for fibrosis and progression of CKD. As MRA were shown to antagonize MBG effects, increased MBG concentrations may further help to identify individuals benefitting most from MRA treatment.

We have to acknowledge that in our study the relatively small sample size of patients with MBG measurement and follow-up as well as restricted clinical data at follow-up decrease the power of or study and limit further discrimination of results. Our findings should therefore be rather regarded as hypothesis generating warranting confirmation in larger well characterized cohorts. To some extent, our results could be influenced by increased prevalence of PA with increasing MBG concentrations, as we have already showed that MBG is higher in patients with PA.[15] Although we could not observe a correlation of plasma MBG concentrations and GFR decline in patients with PA, we must state that these patients underwent specific therapies in the form of adrenalectomy and MRA therapy, that occupied MBG binding sites.[18] We can therefore not fully exclude that PA-specific therapies have partially obscured quantities of GFR decline at follow-up. Due to the setting of the GECOH study as a prospective study for the screening of secondary causes of hypertension at our outpatient clinic it was not possible to hold salt intake of study participants fixed or apply uniformed medical treatment. Unfortunately, study participants did not fill out questionnaires on salt intake and no investigations on body fluid volume expansion were performed, but we obtained spot urine samples at baseline at the first and 24 hour urine samples at a second baseline visit. While not a true substitute for fixed salt intake, we may roughly estimate patients’ short-term salt consumption by urine sodium excretion from spot urine samples. While results suggest there is a common pathway for sodium excretion and MBG (i.e. inflation of beta), sodium excretion surrogates may not explain full variance of the association between MBG and decline in GFR, at least in the short-term. However, one of the main strengths of the GECOH study is that it has been performed within a general hypertensive patient population under ongoing antihypertensive treatment that remained mostly unchanged from general practitioners’ prescriptions (except for spironolactone, canrenoate, eplerenone, amilorid, and/or triamterene which were stopped ≥ 4 weeks before study inclusion). While our limited data on antihypertensive treatment at follow-up preclude further insights, adjustment for intake of different classes of antihypertensives (except for MRA) the association between MBG and decline in GFR remained partially stable in regression analyses. Finally, the observational nature of our study, and some limitations regarding the characterization of kidney status (e.g. missing measurement of albuminuria at follow-up) as well as hard endpoints of kidney disease complicate further interpretation. Our work is only a secondary outcomes analysis of the GECOH study and our data were derived from a Caucasian cohort of hypertensive patients. Our findings may therefore not be generalizable to other study populations.

While our study suggests increased MBG concentration as a predictor of GFR decline, further studies are necessary to validate our results. Additional investigations are warranted to see whether MRA therapy can prevent GFR decline in association with increased MBG concentrations.

## Conclusions

Our data support the hypothesis that a high MBG concentration is associated with markers of kidney impairment. Patients with highest MBG concentrations may be at increased risk of future GFR decline. Whether elevated MBG concentrations predict hard renal endpoints warrants further investigations.

## Supporting information

S1 Dataset Dataset as xlsx file from the GECOH Study. Data from baseline visit(s) and follow-up.
(XLSX)
